# Fatal Accident to a Senior Physician

**Published:** 1912-09-21

**Authors:** 


### Fatal Accident to a Senior Physician.

A very well-known and extremely able practitioner, Dr. J. Ranking, of Tunbridge Wells, was killed last week in a motor-car accident at Bexhill. An Oxford and St. Bartholomew's man, Dr. Ranking was senior physician to the Tunbridge Wells General Hospital, and was highly esteemed as a clinician, not merely by his own patients but also by the practitioners of the neighbouring district, who frequently called him into consultation. He had also been president of the South-Eastern Branch of the British Medical Association. He was, besides an M.D. of Oxford, a Fellow of the Royal College of Physicians, a very unusual distinction for a general practitioner in a town of the size of Tunbridge Wells. It appeared at the inquest that the car in which Dr. Ranking was travelling skidded owing to the slippery surface of the road, turned round twice, ran into a tree, and overturned.

He was elected a physician of the Tunbridge Wells General Hospital in the year 1880, and was a keen member of the committee of management. Dr. Ranking was a Freemason, member of the Homesdale Lodge, and was Worshipful Master in the year 1882. The funeral took place last Tuesday. Besides members of the family, there were present all the members of the hon. medical and surgical staff of the General Hospital who were in the town, and also many other members of the medical profession in Tunbridge Wells and the surrounding districts. The Secretary of the Hospital, Mr. J. J. Webb, attended to represent the chairman and committee, many members of which also were present. The Matron represented the nursing staff. The Freemasons had a deputation, and the local Association of Pharmacists were fully represented.

